# Long Noncoding RNA IGFBP7-AS1 Promotes Odontogenesis of Stem Cells from Human Exfoliated Deciduous Teeth via the p38 MAPK Pathway

**DOI:** 10.1155/2022/9227248

**Published:** 2022-04-16

**Authors:** Dan Wang, Ningxin Zhu, Fei Xie, Man Qin, Yuanyuan Wang

**Affiliations:** ^1^Department of Pediatric Dentistry, School and Hospital of Stomatology, Peking University, Beijing, China; ^2^School and Hospital of Stomatology, Peking University, Beijing, China

## Abstract

Stem cells from human exfoliated deciduous teeth (SHED) are attractive seed cells for dental tissue engineering. Epigenetics refers to heritable changes in gene expression patterns that do not alter DNA sequences. Long noncoding RNAs (lncRNAs) are one of the main methods of epigenetic regulation and participate in cell differentiation; however, little is known regarding the role of lncRNAs during SHED odontogenic differentiation. In this study, RNA sequencing (RNA-seq) was used to obtain the expression profile of lncRNAs and mRNAs during the odontogenic differentiation of SHED. The effect of IGFBP7-AS1 on odontogenic differentiation of SHED was assessed by alkaline phosphatase (ALP) staining, alizarin red S (ARS) staining, quantitative reverse transcription PCR (qRT-PCR), Western blot, and *in vivo*. The level of p38 and p-p38 protein expression was examined by Western blot, and the result was verified by adding the p38 inhibitor, SB203580. The expression profiles of lncRNAs and mRNAs were identified by RNA-seq analysis, which help us to further understand the mechanism in odontogenesis epigenetically. IGFBP7-AS1 expression was increased during odontogenic differentiation on days 7 and 14. The ALP staining, ARS staining, and expression of odontogenic markers were upregulated by overexpressing IGFBP7-AS1 *in vitro*, whereas the expression of osteogenesis markers was not significantly changed on mRNA level. The effect of IGFBP7-AS1 was also verified *in vivo*. IGFBP7-AS1 could further positively regulate odontogenic differentiation through the p38 MAPK pathway. This may provide novel targets for dental tissue engineering.

## 1. Introduction

Hard tissue defects in the maxillofacial tissues, including defects of bone tissue caused by tumors and malformations from birth, as well as tooth defects caused by dental caries and dental trauma, usually require bone reconstruction or dental tissue regeneration. However, existing treatment methods are associated with major disadvantages [1–3]. Recently, biological tissue engineering has provided a feasible method for the regeneration of dental hard tissue [4, 5]. There are three basic elements involved in tissue engineering: (1) cells, (2) growth factors/signals, and (3) scaffolds [6]. The selection of seed cells is the basic issue in tissue engineering. Stem cells from human exfoliated deciduous teeth (SHED) are attractive seed cells for tissue engineering. The use of SHED in dental tissue engineering may be advantageous over traditional stem cells for the following reasons: (1) SHED have a higher rate of proliferation *in vitro* than human bone marrow mesenchymal stem cells (hBMSCs) [7] or dental pulp stem cells (DPSCs) [8]; (2) SHED have a greater capacity to form bone and dentine [9]; and (3) SHED are isolated from deciduous exfoliated teeth, the only disposable organ of the body, which is associated with fewer ethical concerns. In addition, SHED are particularly convenient and safe as autologous seed cells for dental tissue engineering in adolescents with mixed dentition who suffer from pulp necrosis or pulpitis. In our previous study, we aimed to use the steroid, betamethasone, to achieve SHED odontogenesis [10]; however, we found that the osteoclast process was also simultaneously activated. Thus, the purpose of our present study is to elucidate the mechanism by which SHED-directed differentiation can be induced to promote odontogenesis.

Epigenetics refers to heritable changes in gene expression patterns that do not alter DNA sequences. What is more, DNA methylation, chromatin remodeling, and noncoding RNAs are the three main methods of epigenetic regulation. Long noncoding RNAs (lncRNAs), the length of which is over 200 bp, are noncoding RNAs and have been reported to exert their epigenetic functions through different ways such as chemically remodeling chromatin [11]. lncRNAs have been suggested to participate in various biological and pathological processes, such as cell differentiation and proliferation, and cancer development [12]. Previous research has reported the lncRNA expression profiles during mesenchymal stem cell osteo/odontogenesis [13, 14]. lncRNA DANCR has been reported to inhibit the odontogenic differential process of DPSCs through the Wnt/*β*-catenin pathway [15]. In contrast, lncRNA H19 has been shown to improve odontogenic differentiation epigenetically via DLX3 methylation in DPSCs [16] or through the MAPK pathway in stem cells from apical papilla (SCAP) [17]. However, the role of lncRNAs is little known during the odontogenic differentiation of SHED.

Insulin-like growth factor-binding protein 7-antisense 1 (IGFBP7-AS1) is an antisense lncRNA located on chromosome 4 in humans, and the antisense transcript of insulin-like growth factor-binding protein 7 (IGFBP7). IGFBP7-AS1 is reported to be significantly associated with overall survival in patients with glioblastoma, and IGFBP7-AS1 knockdown inhibited the function of glioma cells in viability, migration, and invasion [18]. No studies have considered the role of IGFBP7-AS1 in odontogenic differentiation so far.

In the present study, the expression profiles of lncRNA during odontogenesis of SHED were analyzed by high-throughput RNA sequencing (RNA-seq), which help us to further understand the epigenetic mechanism in odontogenesis. IGFBP7-AS1 was identified as the top differently expressed lncRNA during odontogenic differentiation and selected for further study. The role and mechanistic pathway of IGFBP7-AS1 in the odontogenic differentiation of SHED were investigated, and we aimed to identify a new target for improving tooth regeneration using SHED-based tissue engineering.

## 2. Methods

### 2.1. Cell Culture and Odontogenic Differentiation

SHED were kindly provided by Oral Stem Cell Bank operated by Beijing Tason Biotech Co. Ltd. (http://www.kqgxb.com) from children (age from five to seven) and cultured as previously described [10]. Our experiments were consented with the Ethics Committee of the Peking University School and Hospital of Stomatology, Beijing, China (Approval Number: PKUSSIRB-201732003). SHED at stages P3-P6 were used for subsequent experiments. To induce odontogenic differentiation, SHED were exposed to osteogenic media (OM) comprised of 0.01 mM dexamethasone disodium phosphate, 0.1 mM L-ascorbic acid phosphate, and 1.8 mM monobasic potassium phosphate (Sigma-Aldrich, MO, USA) after reaching 70%-80% confluence. The OM was changed every two days.

### 2.2. Alkaline Phosphatase (ALP) Staining

SHED were cultured in 12-well plates (3 × 10^4^ cells/well) with or without OM, and ALP staining was performed on day 7 using an ALP staining kit according to the manufacturer's protocol (CWbiotech, Beijing, China). Briefly, phosphate-buffered saline (PBS) was used to rinse the cultured cells and the cell layer was fixed in 4% paraformaldehyde for 30 min. Then, it was washed with dH_2_O and incubated in an alkaline solution for 10 min at room temperature.

### 2.3. Alizarin Red S (ARS) Staining

SHED were cultured in 12-well plates. When the cells reached 70%-80% confluency, they were exposed to OM and cultured for an additional 14 days. Briefly, the cells were fixed in 4% paraformaldehyde for 15 min and subsequently stained with 0.1% ARS (pH 4.0-4.6) for 20 min. The integrated density was also measured from histochemical slides.

### 2.4. RNA Isolation and Quantitative Reverse Transcription PCR (qRT-PCR)

TRIzol (Invitrogen, Carlsbad, CA, USA) was used to extract the total RNA, and 1 *μ*g of total RNA was converted to cDNA using a PrimeScript RT Reagent Kit (TaKaRa, Shiga, Japan). qPCR was performed as the previous study described [10]. Each genetic analysis was performed in triplicate, and the primers that were used are listed in Table 1.

### 2.5. RNA Sequencing

The RNA samples were prepared using a total of 3 *μ*g of RNA per sample as the input material. The NEBNext Ultra RNA Library Prep Kit for Illumina (NEB, San Diego, CA, USA) was used to generate the sequencing libraries according to the manufacturer's recommendations. Briefly, mRNA was purified from the total RNA using poly-T oligo-attached magnetic beads, and fragmentation was performed using divalent cations under an elevated temperature in NEBNext First Strand Synthesis Reaction Buffer (5x). First-strand cDNA synthesis was performed using a random hexamer primer and M-MuLV reverse transcriptase (RNase H). Second-strand cDNA synthesis was subsequently performed using DNA.

After the adenylation of the 3′ ends of DNA fragments, the NEBNext adaptor with a hairpin loop structure was ligated in preparation for hybridization. The AMPure XP system (Beckman Coulter, Beverly, USA) was used to purify library fragments. Phusion high-fidelity DNA polymerase, universal PCR primers, and index (X) primer were used in the PCR progress. Finally, the AMPure XP system was used to purify the PCR products and the Agilent Bioanalyzer 2100 system (Agilent Technologies, Santa Clara, California, USA) was used to confirm the library quality. After cluster generation, library preparations were sequenced on an Illumina Hiseq platform, and 125 bp-150 bp paired-end reads were generated. The differential expression of lncRNAs with statistical significance was performed using the EdgeR package on R, and cut-offs were established at a fold change > 1.3 and a *p* value < 0.05.

### 2.6. GO Analysis

Gene Ontology (GO) enrichment analysis of differentially expressed genes was implemented using the clusterProfiler R package (Yu, Wang, Han, and He, 2012), in which gene length bias was corrected. GO terms with corrected *p* values < 0.05 associated with differential expressed genes were considered significantly enriched.

### 2.7. RNA Oligoribonucleotides and Cell Transfection

RNA oligoribonucleotides (e.g., small-interfering RNAs (siRNAs) targeting lncRNA IGFBP7-AS1 and siRNA control (siNC)) were purchased from GenePharma (Shanghai, China). The sequences are listed in Table S1. SHED were cultured in 12-well plates prior to transfection. After reaching 60% confluence, the cells were transfected with siRNAs using Lipofectamine 3000 (Invitrogen, Carlsbad, CA, USA).

### 2.8. Lentivirus Infection

EF-1aF/GFP and puromycin lentiviruses were created by GenePharma (Shanghai, China) to induce lncRNA IGFBP7-AS1 overexpression. SHED were transfected with lentiviruses (MOI: 20) to upregulate the level of lncRNA IGFBP7-AS1. Polybrene (5 mg/mL) was used in the lentivirus medium to improve the infection efficiency. In addition, the medium was changed after 8 h. After three days, the infection efficiency was detected using an inverted fluorescence microscope (Olympus, Japan) and verified using real-time PCR.

### 2.9. Western Blot

The protein lysis buffer containing a phosphatase inhibitor (Applygen Technologies Inc., Beijing, China) was used to harvested cells. The cell suspensions were centrifuged at 4°C for 30 min with a speed of 12,000 × g. The BCA Protein Assay (CWBIO, Beijing, China) was used to determine the protein concentration, and each lane was loaded with equal aliquots of the total protein (20 *μ*g). The sample lysates were separated by sodium dodecyl sulfate-polyacrylamide gel electrophoresis (SDS-PAGE) gels and transferred to polyvinylidene difluoride membranes (Millipore, Bedford, MA), blocked in blocking sodium (Beyotime, Shanghai, China) for 1 h, and probed with the following antibodies at 4°C overnight: DSPP (1 : 1000; Santa Cruz Technology, Santa Cruz, CA), DMP1 (1 : 1000; Bioss, Beijing, China), p38, p-p38, and *β*-actin (1 : 10000; Cell Signaling Technology, Beverly, MA, USA). The membrane was incubated at room temperature for 1 h with horseradish peroxidase- (HRP-) conjugated antirabbit immunoglobulin. The protein expression was detected using a Western enhanced chemiluminescence blotting kit (ECL, SOLIBRO, Beijing, China).

### 2.10. Subcutaneous Transplantation

SHED cultured with *α*-minimum essential medium (MEM) supplemented with 10% fetal bovine serum (negative control (NC) group) or with upregulated lncRNA IGFBP7-AS1 (IGFBP7-AS1 group) were detached using trypsin EDTA and resuspended in PBS. 5 × 10^5^ cells were used and mixed with 1 mg hydroxyapatite (HA), then, incubated at 37°C under 5% CO_2_ for 4 h.

For the implantation procedure, six-week-old SCID mice (males, CB17) were used as subcutaneous transplant recipients. Operations were performed using anesthesia achieved by intraperitoneal injection of pelltobarbitalum natricum. Incisions approximately 0.5 cm in length on each side were made on the dorsal surface of each animal. Subcutaneous pockets were created by blunt dissection, and the cells and HA (1 mg) were placed into the pockets. Incisions were closed with surgical sutures. Animals were sacrificed 8 weeks after implantation. For histology, the tissues were stained with hematoxylin and eosin (H&E) or Masson trichrome (Trichrome Stain (Masson) Kit, Sigma-Aldrich). For the immunohistochemistry staining of DSPP and DMP1, a 1 : 100 dilution of antibody was applied.

### 2.11. Statistical Analysis

SPSS21.0 statistical software (IBM Corp., Armonk, NY, USA) was used to perform the statistical calculations. Comparisons between two groups were analyzed using an independent two-tailed Student's *t*-test, and comparisons between more than two groups were analyzed using a one-way analysis of variance (ANOVA) followed by Tukey's post hoc test. All data were expressed as the mean ± standard deviation (SD) of three experiments per group, and *p* < 0.05 was considered to be statistically significant.

## 3. Results

### 3.1. Odontogenic Differentiation of SHED

**Figure 1:**
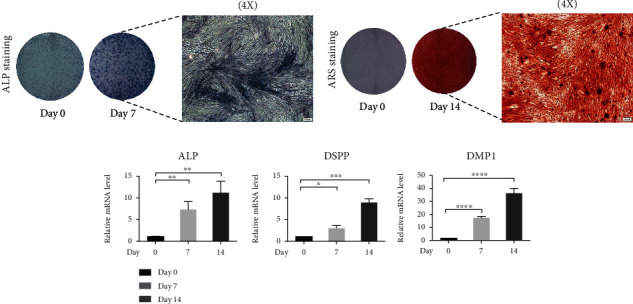
Odontogenic potential of stem cells from human exfoliated deciduous teeth (SHED) during differentiation on day 0, day 7, and day 14. (a) ALP staining of SHED on day 0 and day 7. (b) ARS staining of SHED on day 0 and day 14. Scale bar = 200 *μ*m. ALP: alkaline phosphatase staining; ARS: alizarin red S staining. (c) Expression levels of odontogenic markers ALP, DSPP, and DMP1 are significantly increased during differentiation on day 7 and day 14. Data were expressed as the mean ± standard deviation (SD) of three experiments per group, ^∗∗^*p* < 0.01, ^∗∗∗^*p* < 0.001, and ^∗∗∗∗^*p* < 0.0001.

Odontogenic differentiation of SHED induced by OM was measured by qPCR, ALP staining, and ARS staining. The intensity of ALP staining on day 7 and ARS staining on day 14 is significantly increased (Figure 1(a)). Alkaline phosphatase (ALP) expression is significantly increased on day 7 and day 14 compared with day 0. In addition, the expressions of odontogenic markers, dentin sialophosphoprotein (DSPP) and dentin matrix acid phosphoprotein 1 (DMP1), are significantly increased on day 14 (Figure 1(b)). The total RNA of successfully differentiated cells was subsequently used for RNA-seq.

### 3.2. Expression Profiles of lncRNA and mRNA during Odontogenic Differentiation and Validation

**Figure 2:**
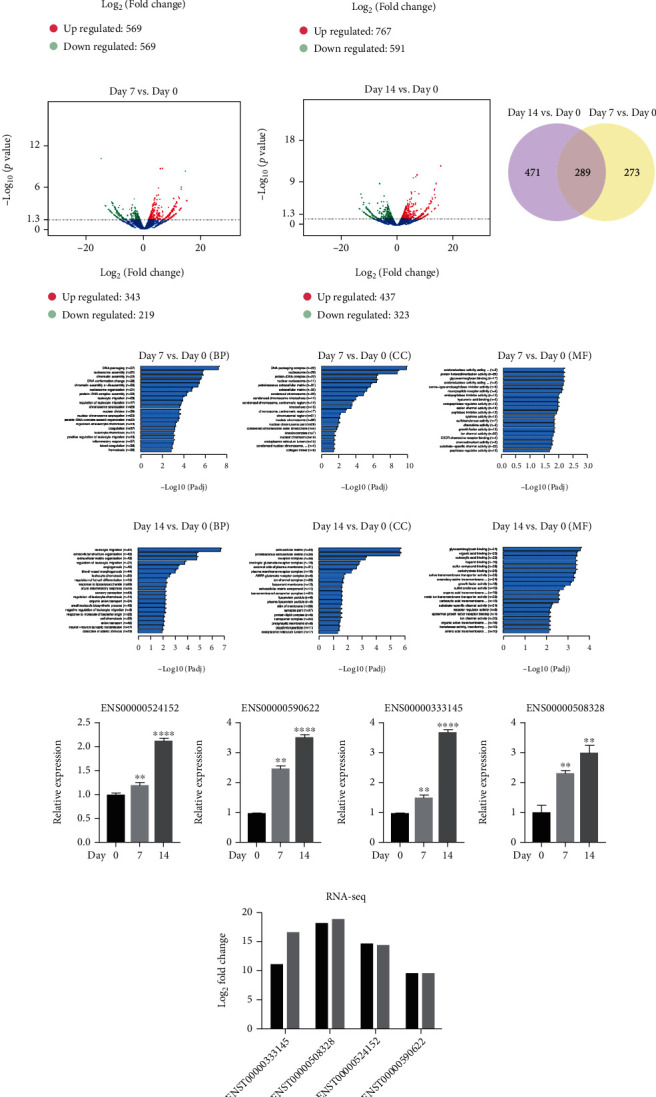
lncRNA and mRNA profiles of stem cells from human exfoliated deciduous teeth (SHED) during odontogenic differentiation. (a) Volcano plot showing 569 lncRNAs upregulated and 569 lncRNAs significantly downregulated on day 7, and 767 lncRNAs upregulated and 591 lncRNAs significantly downregulated on day 14 (Log2 fold change (FC) > 1.3 or ≤-1.3, *p* < 0.05). The Venn diagram shows differentially expressed lncRNAs during odontogenic differentiation of SHED. (b) Volcano plot showing 343 mRNAs upregulated and 219 mRNAs downregulated on day 7, and 437 mRNAs upregulated and 323 mRNAs downregulated on day 14 (Log2 fold change (FC) > 1.3 or ≤-1.3, *p* < 0.05) on gene level. The Venn diagram shows differentially expressed mRNAs during odontogenic differentiation of SHED. (c) Gene Ontology (GO) analysis of mRNAs differentially expressed during odontogenic differentiation in SHED on day 7. (d) GO analysis of mRNAs differentially expressed during odontogenic differentiation in SHED on day 14. (e) qRT-RCR validation of selected lncRNAs differentially expressed during odontogenic differentiation: ENST00000333145, ENST00000508328, ENST00000524152, and ENST00000590622. (f) Results of RNA-sequencing data for selected lncRNAs. Data were expressed as the mean ± standard deviation (SD) of three experiments per group, ^∗^*p* < 0.05, ^∗∗^*p* < 0.01, ^∗∗∗^*p* < 0.001, and ^∗∗∗∗^*p* < 0.0001.

RNA-seq analysis identified 234,832 annotated transcripts of lncRNAs expressed during SHED odontogenic differentiation, of which 136,996 were detected on day 0, 136,832 on day 7, and 138,111 on day 14. There are 1138 lncRNAs differentially expressed on day 7 compared to day 0, whereas 569 lncRNAs are upregulated and 569 lncRNAs are significantly downregulated. In addition, 1358 lncRNAs are differentially expressed on day 14 compared to day 0, among which 767 lncRNAs are significantly upregulated and 591 lncRNAs are significantly downregulated. A Venn diagram analysis reveals that 466 lncRNAs are simultaneously altered on both day 7 and day 14 during odontogenesis (Figure 2(a)).

Subsequent mRNA analysis identifies 1034 mRNAs, among which 343 are upregulated and 219 are significantly downregulated on day 7 compared with day 0, while 437 mRNAs are upregulated and 323 are significantly downregulated on day 14 compared with day 0. Venn diagram analysis reveals that 289 mRNAs are simultaneously altered during odontogenesis (Figure 2(b)).

GO analyses at biological process (BP), cellular component (CC), and molecular function (MF) levels were performed on mRNAs differentially expressed during odontogenic differentiation of SHED. GO analysis of day 7 vs. day 0 data shows that the highest enrichment scores for BP are related to “DNA packaging,” with 27 differentially expressed genes. For CC, the three highest enrichment scores are “DNA packaging complex,” “nucleosome,” and “protein-DNA complex,” associated with 22, 20, and 22 differentially expressed genes, respectively. For MF, “protein heterodimerization activity” has the highest enrichment score and is associated with 29 differentially expressed genes (Figure 2(c)).

GO analysis of day 14 vs. day 0 data reveals that the three highest enrichment scores for BP are “leukocyte migration,” “extracellular structure organisation,” and “extracellular matrix organisation,” which are functionally associated with bone regeneration. For CC, the two highest enrichment scores are “extracellular matrix” and “proteinaceous extracellular matrix,” associated with 43 and 39 differentially expressed genes, respectively. For MF, “glycosaminoglycan binding” has the highest enrichment score (Figure 2(d)).

The characteristics of the top five differently expressed lncRNAs on day 7 and day 14 are listed in Tables 2 and 3. Four candidate lncRNAs (ENST00000333145, ENST00000508328, ENST00000524152, and ENST00000590622) were selected to validate the RNA-seq results, and they exhibit a significant fold change subjected to qRT-PCR analysis (Figure 2(e)). All results are consistent with the normalized RNA-seq data (Figure 2(f)).

### 3.3. The Odontogenic Differentiation of SHED Was Promoted by IGFBP7-AS1

**Figure 3:**
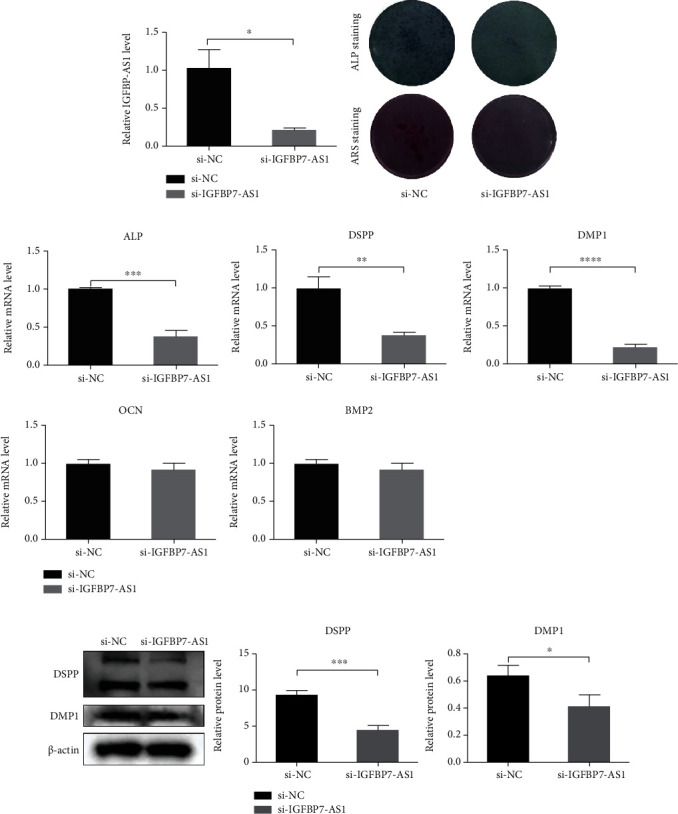
IGFBP7-AS1 knockdown suppresses the odontogenic differentiation of SHED. (a) qRT-PCR detected lncRNA IGFBP7-AS1 levels in the si-NC and si-IGFBP7-AS1 groups. (b) Images of ALP staining of SHED on day 7 after odontogenic differentiation and ARS staining of SHED on day 14 after odontogenic differentiation in the si-NC and si-IGFBP7-AS1 groups. (c) qRT-PCR detected osteo/odontogenic differentiation-related markers: ALP, DSPP, DMP1, OCN, and BMP2 in the si-NC and si-IGFBP7-AS1 groups. (d) Western blot detected odontogenic differentiation-specific markers: DSPP and DMP1 in the si-NC and si-IGFBP7-AS1 groups. Data were expressed as the mean ± standard deviation (SD) of three experiments per group, ^∗^*p* < 0.05, ^∗∗^*p* < 0.01, ^∗∗∗^*p* < 0.001, and ^∗∗∗∗^*p* < 0.0001.

**Figure 4:**
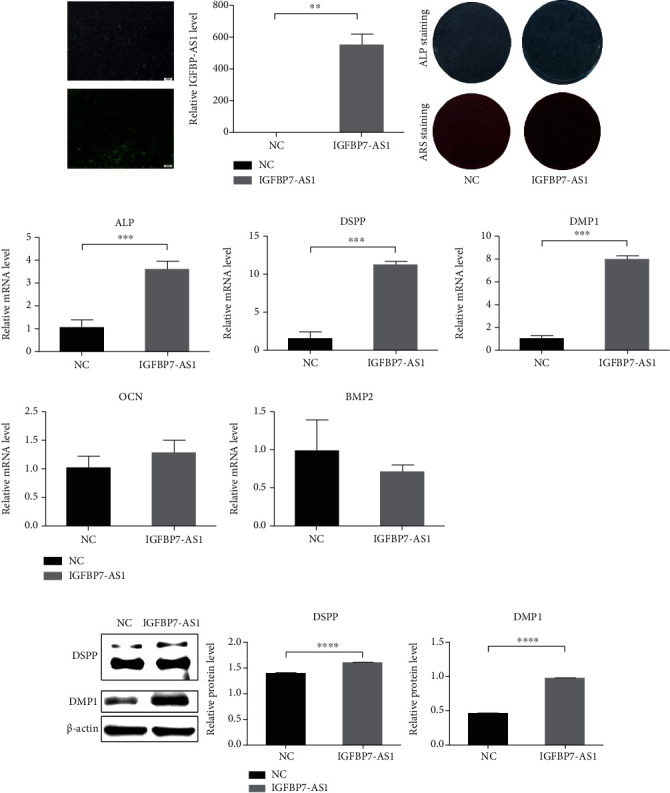
IGFBP7-AS1 overexpression increases the odontogenic differentiation of SHED. (a) Fluorescence microscope and qRT-PCR detected the transfection effect of IGFBP7-AS1 overexpression lentiviruses in the NC and IGFBP7-AS1 groups. Scale bar = 100 *μ*m. (b) Images of ALP staining of SHED on day 7 after odontogenic differentiation and ARS staining of SHED on day 14 after odontogenic differentiation in the NC and IGFBP7-AS1 groups. (c) qRT-PCR detected osteo/odontogenic differentiation-related markers: ALP, DSPP, DMP1, OCN, and BMP2 in the NC and IGFBP7-AS1 groups. (d) Western blot detected odontogenic differentiation-specific markers: DSPP and DMP1 in the NC and IGFBP7-AS1 groups. Data were expressed as the mean ± standard deviation (SD) of three experiments per group, ^∗∗^*p* < 0.01, ^∗∗∗^*p* < 0.001, and ^∗∗∗∗^*p* < 0.0001.

Through RNA sequencing, lncRNA IGFBP7-AS1 is upregulated on both days 7 and 14 during SHED odontogenic differentiation. This result was also confirmed using qRT-PCR. To determine the effect of IGFBP7-AS1 on SHED odontogenic differentiation, lentiviruses were used to overexpress IGFBP7-AS1, and siRNA targeting IGFBP7-AS1 was used to knockdown its expression. The transfection efficiency was observed by a fluorescence microscope, and the expression levels were confirmed using qRT-PCR (Figures 3(a) and 4(a)). SHED successfully transfected were cultured in OM and harvested on days 7 and 14.

The ALP staining results show that the level of ALP expression is decreased by the IGFBP7-AS1 knockdown and increased by IGFBP7-AS1 overexpression after seven days of induction. The ARS staining results reveal that matrix mineralization is reduced in the IGFBP7-AS1 knockdown group and enhanced in the IGFBP7-AS1 overexpression group (Figures 3(b) and 4(b)).

ALP, DSPP, and DMP1 are odontogenic markers, and the expression of them was assessed in SHED cultured in OM for 14 days. IGFBP7-AS1 knockdown significantly downregulates the mRNA level of ALP, DSPP, and DMP1 (Figure 3(c)); however, IGFBP7-AS1 overexpression markedly upregulates the expression of these genes (Figure 4(c)). IGFBP7-AS1 is further confirmed to regulate DSPP and DMP1 at the protein level (Figures 3(d) and 4(d)). Furthermore, the expression of osteogenic differentiation-related markers, BMP2 and OCN, is not significantly altered during this process (Figures 3(c) and 4(c)).

**Figure 5:**
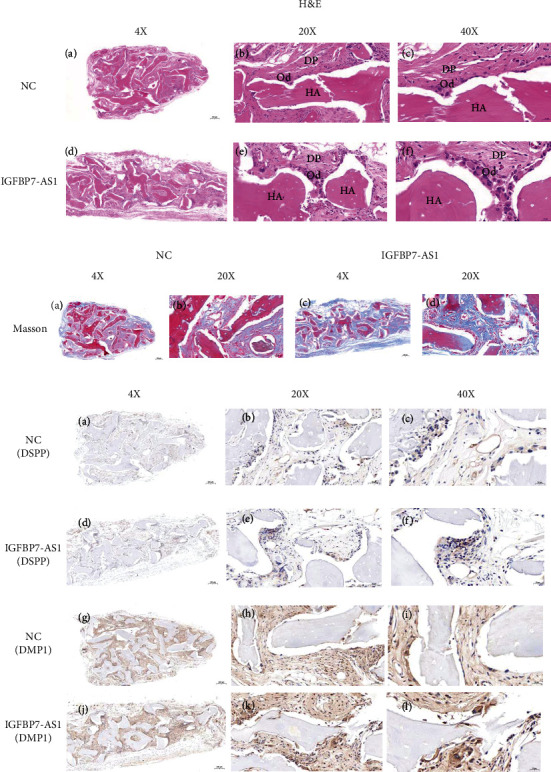
Subcutaneous transplantation results on 8 weeks showing that IGFBP7-AS1 promotes the odontogenic differentiation of SHED *in vivo*. (a) H&E staining, (a–c) NC group, and (d–f) IGFBP7-AS1 group. (b) Masson staining. (c) Immunohistochemistry staining, (a–f) immunohistochemistry staining of DSPP, and (g–l) immunohistochemistry staining of DMP1. Scale bar = 200 *μ*m (4x); scale bar = 50 *μ*m (20x); scale bar = 20 *μ*m (40x) (HA: hydroxyapatite; DP: dental pulp; od: odontoblast).

For the *in vivo* experiments, the H&E results (Figure 5(a)) show that there are more odontoblast-like cells around the materials for the IGFBP7-AS1 group than for the control group. Immunohistochemistry staining of DSPP and DMP1 is consistent with these results (Figure 5(c)). New collagen formation, as revealed by Masson's trichrome staining (Figure 5(b)), is observed for both the NC and IGFBP7-AS1 groups.

### 3.4. IGFBP7-AS1 Regulates the Odontogenic Differentiation of SHED via the MAPK Pathway

**Figure 6:**
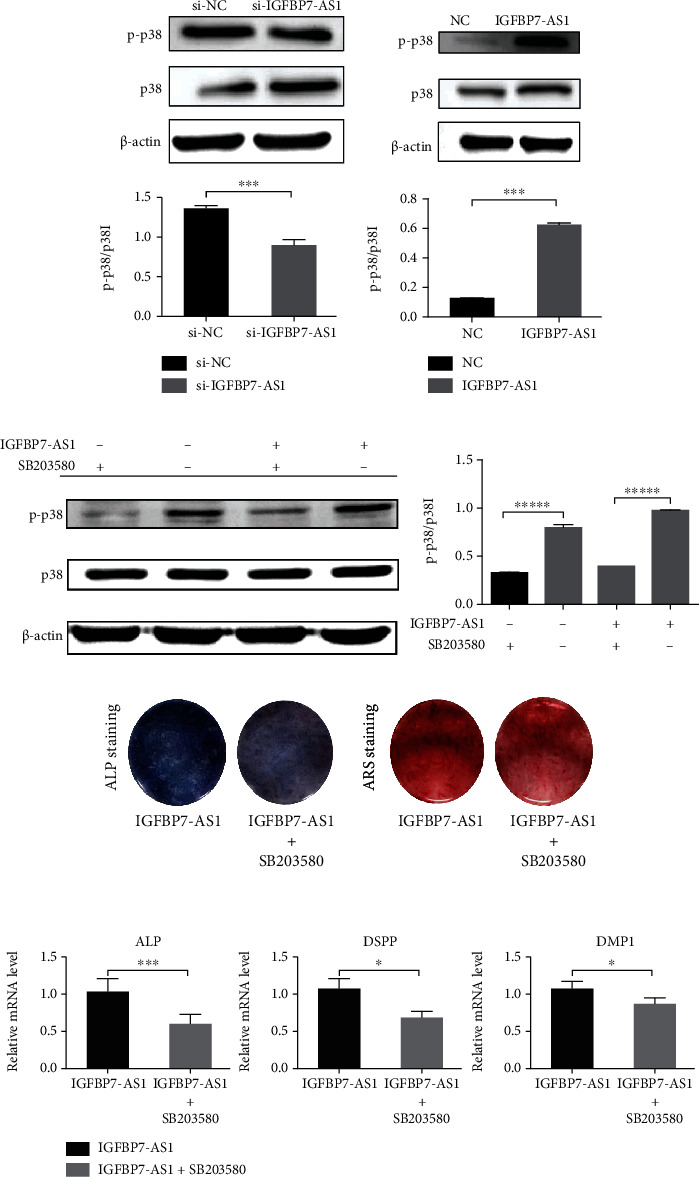
IGFBP7-AS1 regulates the odontogenic differentiation of SHED via the p38 MAPK pathway. (a) Western blot detected the expression of p-p38 and p38 in the si-NC and si-IGFBP7-AS1 groups. The ratio of p-p38 and p38 was calculated. (b) Western blot detected the expression of p-p38 and p38 in the NC and IGFBP7-AS1 groups. (c) Western blot detected the expression of p-p38 and p38 in the NC and IGFBP7-AS1 groups after adding the inhibitor of the p38 MAPK signaling pathway. The ratio of p-p38 and p38 was calculated. (d) Images of ALP staining and ARS staining of IGFBP7-AS1 overexpressed SHED odontogenic differentiation with or without SB203580. (e) qRT-PCR detected odontogenic differentiation-related markers: ALP, DSPP, and DMP1 of IGFBP7-AS1 overexpressed SHED odontogenic differentiation with or without SB203580. Data were expressed as the mean ± standard deviation (SD) of three experiments per group, ^∗^*p* < 0.05, ^∗∗∗^*p* < 0.001, and ^∗∗∗∗^*p* < 0.0001.

Several studies have reported that the MAPK family is a classic pathway that plays a crucial role in the differentiation, mineralization, and proliferation of mesenchymal stem cells [19, 20]. Therefore, in this study, we examined the effects of IGFBP7-AS1 on SHED activation mediated by the p38 MAPK signaling pathway. The level of p-p38/p38 is markedly decreased when IGFBP7-AS1 is knocked down (Figure 6(a)). Importantly, these inhibitory effects are reversed when IGFBP7-AS1 are overexpressed (Figure 6(b)). To validate this result, we also added SB203580, an inhibitor of the p38 MAPK signaling pathway, and the level of p-p38/p38 in the IGFBP7-AS1 overexpressed group is decreased (Figure 6(c)). The ALP staining and ARS staining as well as the expression of odontogenic markers are inhibited by SB203580 in the IGFBP7-AS1 overexpressed group (Figures 6(d) and 6(e)).

## 4. Discussion

Pulp necrosis or pulpitis is a common disease in the dental clinic, which may lead to a loss of dental hard tissue and even tooth loss. These conditions represent a large financial burden to patients and present huge challenges to dentists. Recently, dental tissue engineering has been found to play an important role in resolving these challenges.

Since SHED have the potential for multidirectional differentiation [21], as such, they are considered to be favorable seed cells for tissue engineering due to their ability to develop into neural cells, adipocytes, and osteoblasts [9]. Moreover, they express odontogenesis-related markers (e.g., DSPP and DMP1), and they have been reported to differentiate into odontoblast-like cells *in vivo* [22]. In the present study, we found that SHED possessed the potential to form calcified tissue and to express DSPP and DMP1 induced by osteo/odontogenetic media, similar to the results of previous studies [8]. This finding indicates that SHED may represent a good choice of seed cells for the regeneration of dental hard tissue.

lncRNAs are an important part of epigenetic regulation. Emerging evidence indicates that lncRNAs may play a crucial role in the regulation of differentiation and stem cell biology, and they may also be key regulators involved in human hard tissue regeneration [23, 24]. Previous studies have determined the expression profile of lncRNAs and mRNAs in different types of mesenchymal stem cells during the osteogenic process under various conditions [25–27] and confirmed that some lncRNAs including MALAT1, DANCR, H19, and MIR31HG can regulate osteogenic differentiation through classic osteogenic signaling pathways, including the Wnt/*β*-catenin, mitogen-activated protein kinase (MAPK), nuclear factor kappa-B (NF-*κ*B), and bone morphogenetic protein-2 (BMP2) pathways [28–32]. Osteogenesis shares many similarities with odontogenesis, such as extracellular matrix synthesis. One study explored the differential expression of lncRNAs during the osteogenic/odontogenic differentiation of DPSCs and found that lncRNA SNHG7 may represent a potential target for the osteo/odontoblast differentiation of DPSCs [14]. Furthermore, lncRNA H19 has been reported to promote the odontogenic differentiation and lncRNA DANCR has been reported to inhibit the odontogenic differentiation epigenetically. However, lncRNAs tend to exhibit both strong cell- and tissue-specific expression [33]. Thus, in the present study, we measured the level of lncRNA expression in SHED during odontogenic differentiation using high-throughput sequencing. The expressions of lncRNAs and mRNAs are significantly changed during odontogenic differentiation. These remarkable differences during differentiation indicate that lncRNAs might play a key role in the odontogenic differentiation of SHED, consistent with the findings in other types of mesenchymal stem cells [14]. GO analysis on day 7 reveals that the highest enrichment scores of BP, CC, and MF categories are “DNA packaging,” “DNA packaging complex,” and “protein heterodimerisation activity,” which are all related to epigenetic regulation and indicate epigenetically process may exert a crucial role in this stage. Meanwhile, on day 14, the highest enrichment scores for BP, CC, and MF categories are all related to ECM. Interactions between cells and ECM control various cellular activities including adhesion, differentiation, proliferation, and apoptosis, all of which are associated with odontogenesis [34]. The changes in expression patterns of mRNAs and lncRNAs will help us explore the relationship between them in the future.

The RNA-sequencing results reveal that lncRNA IGFBP7-AS1 was significantly upregulated on both day 7 and day 14. lncRNA IGFBP7-AS1 is an antisense transcript of insulin-like growth factor-binding protein 7 (IGFBP7) and has been reported to be relevant to cancer [18]; however, no study has reported the role of IGFBP7-AS1 on odontogenic differentiation of SHED. Thus, qPCR was used to confirm the reliability of the RNA-sequencing results. The level of IGFBP7-AS1 expression is significantly upregulated during the odontogenic differentiation of SHED, which is consistent with the RNA-sequencing results. By overexpressing IGFBP7-AS1, the levels of ALP activity and mineralized matrix deposition are abnormally increased in the SHED. In contrast, the downregulation of IGFBP7-AS1 generates the opposite results. DSPP and DMP1 are specific markers of odontogenic differentiation used to distinguish osteogenesis [35, 36]. In the present study, the expression of DSPP and DMP1 is upregulated at both the gene and protein level after overexpressing IGFBP7-AS1 and downregulated after IGFBP7-AS1 is knocked down. Interestingly, the levels of osteogenesis-related markers, BMP2 and OCN, remain remarkably unchanged when IGFBP7-AS1 was altered. The effect of IGFBP7-AS1 *in vivo* was also confirmed by subcutaneous transplantation into SCID mice. The results are consistent with our hypothesis that IGFBP7-AS1 plays a key role in the promotion of odontogenesis in SHED. Thus, we confirm the positive effect of IGFBP7-AS1 on the directed odontogenic differentiation of SHED, which may provide a new target for dental tissue regeneration.

Osteogenesis shares many similarities with odontogenesis, such as the secretion of type I collagen and noncollagen proteins and the extracellular matrix mineralization, and they may overlap with a signaling pathway, such as Wnt/*β*-catenin, mitogen-activated protein kinase (MAPK), and bone morphogenetic protein-2 (BMP2) pathways. From our RNA-sequencing results, we found that on day 14, the highest enrichment scores of GO analysis are all related to extracellular matrix, which is important in various cellular activities including adhesion, differentiation, and proliferation. The MAPK signaling pathway is important to the osteo/odontogenic differentiation of mesenchymal stem cells, which includes the p38, JNK, and ERK1/2 pathways. Previous studies have reported that the MAPK pathway regulates the extracellular matrix mineralization and plays a crucial role in triggering osteo/odontogenic differentiation [37–39]. Thus, we used the MAPK pathway as a candidate pathway. In the present study, the level of p-JNK and p-ERK1/2 is not influenced by IGFBP7-AS1 (data was not shown), whereas the level of p-p38 protein expression is eliminated when IGFBP7-AS1 is knocked down and reversed by the overexpression of IGFBP7-AS1. When adding the inhibitor of the p38 MAPK signaling pathway, the increase effect of IGFBP7-AS1 in odontogenesis of SHED is impaired. These findings revealed that IGFBP7-AS1 regulates the odontogenic differentiation of SHED by promoting p38 activation to p-p38 and promoting the expression of odontogenic differentiation-related markers: ALP, DSPP, and DMP1.

## 5. Conclusion

In conclusion, we determined the lncRNA and mRNA expression profiles of SHED during odontogenesis and the role of IGFBP7-AS1 in this process. The results indicate that lncRNAs may represent important regulators of odontogenic differentiation in SHED and the expression profiles help us to further understand the epigenetic mechanism in odontogenesis. In addition, IGFBP7-AS1 may promote the odontogenic differentiation of SHED through the MAPK pathway. Although these findings provide new potential targets for dental tissue engineering, the specific molecular mechanism of IGFBP7-AS1 is needed in the future.

## Figures and Tables

**Table 1:** Sets of primers used in qPCR.

Gene name		5′-3′	Size (bp)	Gene bank number
GAPDH	F	CCGTCTTGAGAAACCTGCCA	139	NM_001115114.1
	R	GGATGAACGGCAATCCCCAT		

ALP	F	CTCCATACCTGGGATTTCCGC	299	NM_000478.6
	R	GGCCCCAGTTTGTCCTTCTT		

DSPP	F	GGAATGGCTCTAAGTGGGCA	284	NM_014208.3
	R	CTCATTGTGACCTGCATCGC		

DMP1	F	GAGTGGCTTCATTGGGCATAG	260	NM_004407.4
	R	GACTCACTGCTCTCCAAGGG		

OCN	F	TCACACTCCTCGCCCTATTG	133	NM_199173.6
	R	CTCTTCACTACCTCGCTGCC		

BMP2	F	ACTCGAAATTCCCCGTGACC	144	NM_001200.4
	R	CCACTTCCACCACGAATCCA		

ENST00000333145	F	GGCTTTGGGTATGGCTAAT	89	NR_015377.2
	R	AAGGTTCTGGAAGGTTGC		

ENST00000508328	F	GGTTGGGTTCATGTGCTAC	121	NR_034081.1
	R	AGAATTGCTTCCTGCTAATCT		

ENST00000524152	F	GGCAACAACAGTCTTCTATCC	105	NR_033651.1
	R	TGCTGCCCTTTATTGTGCTA		

ENST00000590622	F	TTTCTCATCCGTCCACCG	94	NR_038278.1
	R	CGTACCTTTAATCTGGAGACAA		

**Table 2:** Characteristics of the top five differently expressed lncRNAs on day 7.

Gene symbol	Sequence name	Regulation	Chromosome	Log2 fold change	*p* value
IGFBP7-AS1	ENST00000508328	Up	Chr4	18.1404343	0.00109902
ZBTB16	ENST00000541602	Up	Chr11	16.6554103	1.27*E* − 05
RPL4	ENST00000561775	Down	Chr15	-19.561437	0.00072486
ADAMTS9-AS2	ENST00000481312	Down	Chr3	-16.138993	2.46*E* − 05
LINC00511	ENST00000649793	Down	Chr17	-15.747108	0.00295472

**Table 3:** Characteristics of the top five differently expressed lncRNAs on day 14.

Gene symbol	Sequence name	Regulation	Chromosome	Log2 fold change	*p* value
IGFBP7-AS1	ENST00000508328	Up	Chr4	18.76836388	1.69*E* − 05
ZBTB16	ENST00000541602	Up	Chr11	18.17336972	1.75*E* − 06
PAX8-AS1	ENST00000333145	Up	Chr2	16.7385569	0.00176779
SH3BGRL2-OT1	TCONS_00271625	Up	Chr6	16.27896439	0.002127
RPL4	ENST00000561775	Down	Chr15	-19.531867	0.00064332

## Data Availability

The data that support the findings of this study are available from the corresponding author upon reasonable request.

## Supplementary Materials

Supplementary Materials Table S1 Sequences of siRNA targeting lncRNA IGFBP7-AS1.
