# Supplementary material for: Long Noncoding RNA IGFBP7-AS1 Promotes Odontogenesis of Stem Cells from Human Exfoliated Deciduous Teeth via the p38 MAPK Pathway
Source: Stem Cells Int. 2022 Apr 16;2022:9227248. doi: 10.1155/2022/9227248 (PMC9034958; doi:10.1155/2022/9227248)
Supplement: Supplementary Materials — Table S1 Sequences of siRNA targeting lncRNA IGFBP7-AS1. [file 9227248.f1.docx]

**Supplementary material**

Table S1 Sequences of siRNA targeting lncRNA IGFBP7-AS1

|  | sense（5'-3'） | antisense（5'-3'） |
| --- | --- | --- |
| si-IGFBP7-AS1 | GCUCUUCCUGACCCACAAA | UUUGUGGGUCAGGAAGAGC |
